# MassiveFold: unveiling AlphaFold’s hidden potential with optimized and parallelized massive sampling

**DOI:** 10.1038/s43588-024-00714-4

**Published:** 2024-11-11

**Authors:** Nessim Raouraoua, Claudio Mirabello, Thibaut Véry, Christophe Blanchet, Björn Wallner, Marc F. Lensink, Guillaume Brysbaert

**Affiliations:** 1 Université de Lille, CNRS, UMR 8576 - UGSF - Unité de Glycobiologie Structurale et Fonctionnelle, Université de Lille, CNRS, Lille, France; 2 Science for Life Laboratory, Department of Physics, Chemistry and Biology, National Bioinformatics Infrastructure Sweden, Linköping University, Linköping, Sweden; 3 Institut du Développement et des Ressources en Informatique Scientifique (IDRIS), CNRS, Université Paris-Saclay, Orsay, France; 4 IFB-core, Institut Français de Bioinformatique (IFB), CNRS, INSERM, INRAE, CEA, Evry, France; 5 Division of Bioinformatics, Department of Physics, Chemistry and Biology, Linköping University, Linköping, Sweden

**Keywords:** Protein structure predictions, Machine learning, Software

## Abstract

Massive sampling in AlphaFold enables access to increased structural diversity. In combination with its efficient confidence ranking, this unlocks elevated modeling capabilities for monomeric structures and foremost for protein assemblies. However, the approach struggles with GPU cost and data storage. Here we introduce MassiveFold, an optimized and customizable version of AlphaFold that runs predictions in parallel, reducing the computing time from several months to hours. MassiveFold is scalable and able to run on anything from a single computer to a large GPU infrastructure, where it can fully benefit from all the computing nodes.

## Abstract (web summary)

Although AlphaFold is very efficient for protein structure prediction, massive sampling is a very GPU demanding task. MassiveFold overcomes this limitation, being capable of parallelizing structure prediction computation.

## Main

The protein structural space has been rendered substantially more accessible with DeepMind’s AlphaFold and the AlphaFold Protein Structure Database^1–3^. AlphaFold was originally trained and parameterized on and for single protein chains only, but it has since been retrained for multimer applications^4^. The recent CASP15-CAPRI round of blind structure prediction has shown widespread use of its inference engine in the modeling of protein assemblies, with notable success^5,6^. After demonstrating a proof of concept with the application of massive AlphaFold sampling to protein–peptide interaction^7^, the AFsample tool was successfully applied to the modeling of protein complexes, including difficult-to-model nanobody complexes^8,9^, and ranked first in the CASP15-CAPRI assembly modeling category^5^. Very recently, it was shown that the massive sampling approach can also be applied to such specific binding as antigen–antibody interactions^10^. For monomeric structures too, increasing the sampling can help in the investigation of conformational variability^11^. In addition, it has become evident that increasing the number of recycles in the process may also lead to an improvement in the quality of prediction^12^, but at the cost of prolonged computing times for every single prediction. As a whole, the application comes with a high cost as it cannot run in parallel and is very greedy in terms of graphics processing unit (GPU) resources and time, making it impractical to run even for dedicated research teams.

The computing infrastructures that host GPU clusters and provide resources for such high computing demands often carry restrictive job walltimes due to the high demand on these clusters, preventing prolonged AlphaFold calculations. For large assemblies, it may even be that these walltimes prevent the conclusion of a ‘standard’ AlphaFold-Multimer run of 25 predictions.

In this Brief Communication we present MassiveFold, which combines the framework of AlphaFold^1^ with the enhanced sampling of AFsample^8^ and the added functionality from ColabFold^12^. MassiveFold is a parallelization engine that calls the structure prediction tool, which can be AFmassive, an extended version of AFsample that we developed alongside MassiveFold, or ColabFold, and then performs a post-treatment on the results. Other structure prediction engines can be integrated into MassiveFold in the future, provided they are massive sampling enabled. MassiveFold includes all versions of neural network (NN) models released by AlphaFold so far, contains multiple parameters that lead to an increase in structural diversity (a full list is provided in the Methods), and can be instructed to keep only the results of the most promising predictions. The program can run many instances in parallel, down to a single prediction per GPU, therefore making optimal use of the available computing infrastructure and allowing a substantial reduction in time required to obtain prediction results, from several months to hours. MassiveFold is easy to install through a conda environment and is easy to use, running a simple command line with a JavaScript Object Notation (JSON) parameter file.

**Fig. 1:**
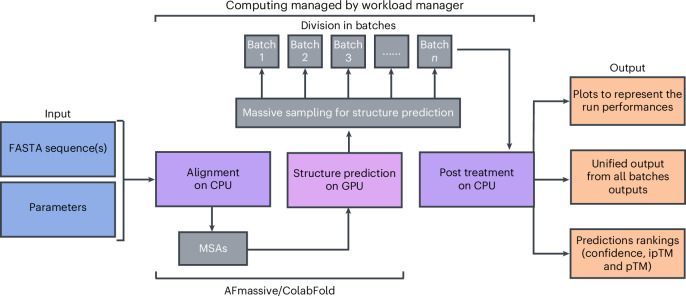
Computing processes handled automatically by MassiveFold. The provided inputs are the FASTA sequence(s) and parameter options for AFmassive or ColabFold. MassiveFold then runs the alignments on a CPU, producing multiple sequence alignments (MSAs) and divides the structure predictions for massive sampling in batches to be run on GPUs. After completion, MassiveFold automatically gathers all predictions, ranks them following the AlphaFold ranking confidence score, the predicted template modeling score (pTM) and interface predicted template modeling score (ipTM), and generates plots.

To enable full access to the diversity parameters, MassiveFold integrates an optimized parallelization that consists of three parts (Fig. 1): (1) alignments computation on a central processing unit (CPU), (2) a structure inference split into many batches on GPUs and (3) a final post-processing step on a CPU that gathers the results, ranks all the predictions, and generates plots (details are provided in the Methods).

**Fig. 2:**
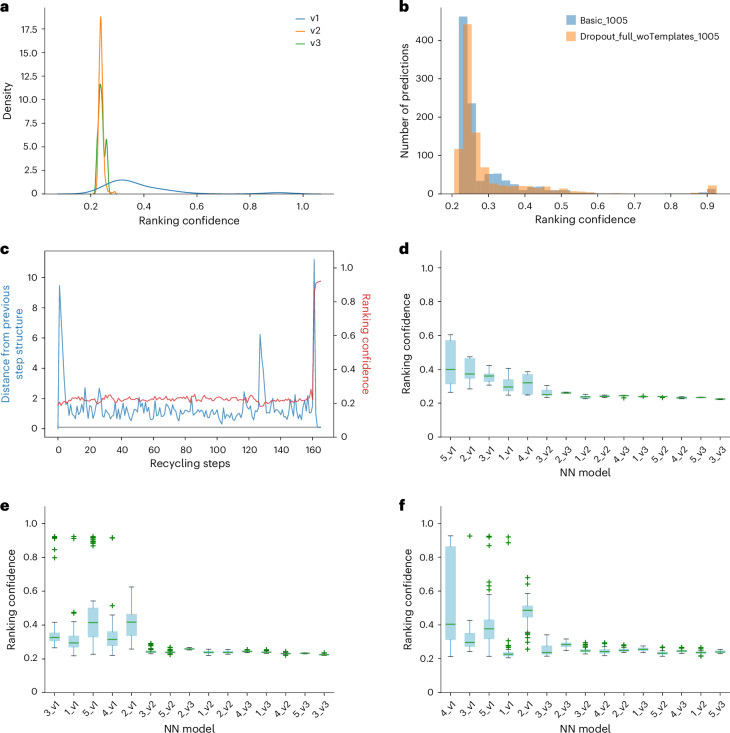
Plots generated by MassiveFold using AFmassive for structure prediction for CASP15 multimeric target H1140. **a**, Ranking confidence density for each of the three NN versions currently available, running 67 predictions per NN model, with default parameters, that is, without diversity parameters activated. **b**, Ranking confidence distributions for two sets of parameters, running 67 predictions per NN model. **c**, Recycle plot of a single prediction, with an early-stop tolerance set to 0.1 and at most 1,000 recycling steps performed. Ranking confidence is shown in red and the distance to the previous structure in blue. As this distance fell below the early-stop tolerance (shown in gray), early stop was triggered at step 164. **d**–**f**, Boxplots of the ranking confidence for each NN model (15 for multimers, five for monomers) sorted by the highest ranking confidence: five predictions per NN model, default parameters (**d**), 67 predictions per NN model, default parameters (**e**), 67 predictions per NN model, activating dropout in the Evoformer and structure modules, and not using templates (**f**). Each box in cyan extends from the first quartile to the third quartile, with a green line at the median, the whiskers reach out to the furthest data point within 1.5 times the interquartile range from the box, and outliers (green crosses) lie beyond the whiskers. Source data

The post-processing of MassiveFold assembles all prediction results and produces several plots. These include the well-known predicted local distance difference test (pLDDT) and predicted aligned error (PAE) plots following AlphaFold and/or ColabFold coloring schemes (Supplementary Fig. 2a,b), but also the ColabFold alignment depths plots (Supplementary Fig. 2b), even if ColabFold was not selected as the inference engine. In addition, MassiveFold plots the distribution of confidence scores per AlphaFold NN version (Fig. 2a), per individual NN model (Fig. 2d–f) or all together (Supplementary Fig. 3). Because MassiveFold can be run with different parameter sets, a plot to compare the distribution of confidence scores between these sets can also be generated (Fig. 2b). The final plot shows the evolution of the AlphaFold confidence score over the recycling and the distance between consecutive structures, which is to be compared to the early-stop-tolerance parameter (Fig. 2c).

The score distribution and recycle plots show the prediction behavior, as they highlight diversity in the AlphaFold confidence score as a function of the applied NN model. Figure 2d shows the diversity in the predictions for a default run of MassiveFold for CASP15 target H1140 (ref. ^6^). Here, 75 structures were generated (five per NN model) with a highest score not exceeding 0.6. Extending the calculation to 1,005 structures (67 per NN model, no other changes in parameters) already produces a few outliers with confidence scores above 0.8 (Fig. 2e). This distribution can even be improved by activating the dropout and excluding templates (Fig. 2f), demonstrating the added value of the massive sampling strategy. The figures also show that in this instance, the v1 NN model was the only model that produced high-confidence structures, and the computing time could have been reduced by only extending the sampling of the five first NN models (all v1).

An additional approach to massive sampling is through the recycling parameters, which play a non-negligible role in diversity generation. Figure 2c shows the recycling behavior of a structure prediction for CASP target H1140, using AFmassive with dropout and without templates and an early-stop tolerance of 0.1, while allowing up to 1,000 recycling steps. The figure shows low confidence scores for the first 160 recycling steps, which then suddenly jump to 0.846 and 0.908. With an early-stop tolerance of 0.5, only four of the ten best predictions show this jump, as opposed to all ten for an early-stop tolerance of 0.1 (Supplementary Table 1). One therefore has to consider extensive recycling as a viable alternate complementary to massive sampling. Splitting the computing with MassiveFold allows easy access to such an exploration.

MassiveFold can use either AFmassive or ColabFold as inference engines. In both cases, however, outliers with high confidence scores will only be generated by using a massive sampling strategy with diversity parameters activated (Fig. 2 and Supplementary Fig. 4).

Recently, DeepMind published AlphaFold3^13^, an all-in-one tool for structure predictions of biomolecular interactions, for which the authors claim it improves protein–protein complex predictions compared to their latest release, AlphaFold2.3, in particular for antibody–antigen predictions. To show the added value of using massive sampling via MassiveFold, we computed predictions with AlphaFold3 for the six CASP15 targets highlighted in Wallner’s massive sampling manuscript^9^ and for two additional CASP15 antibody–antigen targets for which massive sampling produced better models that were not recognized as such^9^. Supplementary Table 2 shows that AlphaFold3 only marginally outperforms massive sampling for three of the eight targets, whereas MassiveFold produces good models for seven of them. For the remaining target (H1167) neither approach produces acceptable models. However, the main advantage of AlphaFold3 is that it produces a more reliable score than AlphaFold2 for antibody–antigen targets, which fails to score these predictions efficiently, as demonstrated in refs. ^9,14^. Depending on the target, either MassiveFold or AlphaFold3 may produce the best models, highlighting the benefit of having AlphaFold3 integrated into MassiveFold, which we intend to do, should the code be released by the authors.

MassiveFold was designed to facilitate access to diversity parameters and to optimally manage the computing. It takes full advantage of a GPU cluster for the inference step, while using a CPU for the multiple sequence alignment and post-processing, which do not require a GPU. It is also optimized for use on a single GPU machine, because massive sampling jobs can be run in low priority, thus allowing higher-priority jobs to insert themselves into the computing queue. MassiveFold is ready for a massive exploration of the AlphaFold protein structure prediction landscape.

## Methods

MassiveFold was developed in bash and Python 3. MassiveFold v1.2.5 integrates the optimized parallelization into CPU and GPU jobs, including post-processing for reranking and plot generation (Fig. 1). The user can select either AFmassive v1.1.5 or ColabFold v1.5.5^12^ for structure inference, both of which are included in the MassiveFold distribution.

AFmassive was developed in Python 3. It is an extended version of AFsample^8^ based on AlphaFold v2.3.2. It integrates all versions of the AlphaFold NN models currently available, that is, one for monomers and three for multimers, and includes additional parameters (listed in Supplementary Notes). These parameters can be set in the AFmassive JSON parameter file (ColabFold JSON parameter file for ColabFold).

### Diversity parameters

The diversity parameters included in MassiveFold are the following: all NN models released by AlphaFold so far (including previous versions, that is, five for monomers and 15 for multimers), the activation of the dropout in the EvoFormer module and the structure module, the use of templates, and the number of recycle steps and the early-stop tolerance threshold, with the recycling stopping if the distance between the current and preceding structure falls below this threshold. In addition, MassiveFold accepts an additional JSON file as input, specifying individual dropout rates (Supplementary Fig. 1 presents a list of rates), thereby providing the user with additional options to increase structural diversity.

### MassiveFold process

MassiveFold v1.2.5 integrates parallelization based on the Simple Linux Utility for Resource Management (SLURM) workload manager. Input given on the command line includes a FASTA file with protein sequence(s), a JSON parameter file, the inference engine to use (AFmassive or ColabFold so far), and the desired number of predictions per NN model, divided into batches of automatically calibrated or manually set size. An example of the JSON parameter file is provided. It contains the parameters for the computing infrastructure and individual runs, including, most importantly, the diversity parameters. The autocalibration adapts the batch size following an initial basic run of MassiveFold (for example, with five predictions per NN model) to keep the process duration under the walltime. The maximum prediction time will be used in comparison with the specified walltime to automatically calculate the number of batches. The minimum number of batches is the number of NN models used.

Once these parameters are set, the pipeline is as follows (Fig. 1): (1) the multiple sequence alignments running on CPU cores; (2) the structure inference processing each batch of calculations on a single GPU core, ensuring that the number of GPU cores used corresponds to the number of batches to run; (3) the post-processing running on CPU cores, to gather and rank the predictions (following AlphaFold metrics), and to generate plots.

In step (1), the alignments are either performed with JackHMMer and HHblits when using AFmassive, or MMseqs2 when using ColabFold. In step (2), the structure inference is either performed by AFmassive or ColabFold. In step (3), if ColabFold is used, outputs are converted to AlphaFold’s output format: structure file names are prefixed by its ranking index, ranking_debug.json file is created and pickle file names are reformatted. AFmassive uses this format natively. In both cases, a ‘light’ pickle option is available, which substantially reduces the size of the pickle files while keeping the main information. Steps (2) and (3) only start once the previous step is completed. It should be noted that it is possible to use pre-computed alignments by putting them in the output folder. They will be detected and not computed again unless a recalculation is forced.

In addition, a gather_runs.py script is provided to let the user collate several runs of MassiveFold. It gathers all the predictions and ranks them. This was used during the CASP16 MassiveFold generation allowing a consolidated ranking over all eight applied run conditions (including ranking_debug.json, pdb and pickle files), for a total of up to 8,040 predictions per CASP16 target.

### Calculation

All inference calculations were performed on V100 or A100 GPUs. The five sets of parameters used for the massive sampling generation of predictions with AFmassive are listed in the Supplementary Notes, as well as the two sets of parameters used for ColabFold.

### Reporting summary

Further information on research design is available in the Nature Portfolio Reporting Summary linked to this Article.

## Supplementary information

Supplementary Information Supplementary Notes, Figs. 1–4 and Tables 1 and 2.

Reporting Summary

Supplementary Data 1 Data for Supplementary Figs. 3 and 4a,b.

## Source data

Source Data Fig. 2 Statistical source data for Fig. 2.

## Data Availability

CASP15 target H1140 was used as a case study, and the sequence for this is available at https://predictioncenter.org/casp15/targetlist.cgi and corresponds to PDB structure 9ERT. All sequences in Supplementary Table 2 are also available at https://predictioncenter.org/casp15/targetlist.cgi. Source data are provided with this paper.

## Code availability

The code for MassiveFold is publicly available at https://github.com/GBLille/MassiveFold, and release v1.2.5 is also available in Zenodo at 10.5281/zenodo.13870060 (ref. ^15^). The code for AFmassive is publicly available at https://github.com/GBLille/AFmassive and release v1.1.5 is also available in Zenodo at 10.5281/zenodo.13869932 (ref. ^16^). A conda environment is provided in their respective repository for each installation. The installation of ColabFold was performed with the ColabFold v1.5.5 conda environment accessible at https://anaconda.org/bioconda/colabfold. MassiveFold conda installation includes the installation of AFmassive and ColabFold. MassiveFold is under the CeCILL v2.1 license.
