# Supplementary material for: MassiveFold: unveiling AlphaFold’s hidden potential with optimized and parallelized massive sampling
Source: Nat Comput Sci. 2024 Nov 11;4(11):824–8. doi: 10.1038/s43588-024-00714-4 (PMC11578886; doi:10.1038/s43588-024-00714-4)
Supplement: Supplementary file 1 — Supplementary Notes, Figs. 1–4 and Tables 1 and 2. [file 43588_2024_714_MOESM1_ESM.pdf]

nature computational science

Supplementary information https://doi.org/10.1038/s43588-024-00714-4

MassiveFold: unveiling AlphaFold's hidden potential with optimized and parallelized massive sampling

In the format provided by the authors and unedited

Supplementary information

Table of contents

Supplementary Notes......1
Supplementary Figures and Tables......3
References......5

Supplementary Notes

MassiveFold v1.2.5 parameters that can be specified in the JSON parameter file:
"models_to_use": list of neural network models to use; by default all are used
"pkl_format": how to manage pickle files

- 'full' to keep the pickle files generated by the inference engine,
- 'light' to reduce its size by selecting main components, which are: number of recycles, PAE values, max PAE, plddt scores, ptm scores, iptm scores and ranking confidence values (stored in ./light_pkl directory)
- 'none' to remove them

Parameters added to AlphaFold v2.3.2 in AFmassive v1.1.5 and used with *run_AFmassive.py*:
--*dropout_rates_filename*: provides dropout rates at inference from a JSON file.
--*early_stop_tolerance*: early stop threshold for recycling
--*bfd_max_hits*: max hits in BFD/uniref MSA
--*mgnify_max_hits*: max hits in mgnify MSA
--*uniprot_max_hits*: max hits in uniprot MSA
--*uniref_max_hits*: max hits in uniref MSA
--*start_prediction*: prediction to start with, can be used to parallelize jobs
--*end_prediction*: prediction to end with, can be used to parallelize jobs
--*stop_recycling_below*: after the first recycle step, only predictions with ranking confidence above this score will continue recycling; predictions below this threshold will still be present in *ranking_debug.json* and produced output
--*min_score*: predictions with a score below this threshold will be excluded from the output
--*max_score*: terminates the computing process when a suitable prediction with a ranking confidence > max_score has been obtained
These parameters are accessible and can be used like the other flags of AlphaFold through the *run_AFmassive.py* script (instead of *run_alphafold.py*). All parameters will be listed with *run_AFmassive.py --help*. In the context of MassiveFold v1.2.5, these parameters can be set in the AFmassive JSON parameters file under the "AFM_run" section.

Parameters of ColabFold v1.5.5 (as described by the authors) that can be set in the ColabFold JSON parameters file for MassiveFold v1.2.5 and that refer to the *colabfold_batch* executable (details accessible through *colabfold_batch --help* except "model_preset" which was added):
"model_preset": multimer or monomer_ptm
"pair_strategy": How sequences are paired during MSA pairing for complex prediction. complete: MSA sequences should only be paired if the same species exists in all MSAs. greedy: MSA sequences should only be paired if the same species exists in at least two MSAs. Typically, greedy produces better predictions as it results in more paired sequences. However, in some cases complete pairing might help, especially if MSAs are already large and can be well paired.
"use_dropout": Activate dropouts during inference to sample from uncertainty of the models. This can result in different predictions and can be (carefully!) used for conformations sampling.
"num_recycle": Activate dropouts during inference to sample from uncertainty of the models. This can result in different predictions and can be (carefully!) used for conformations sampling.

"recycle_early_stop_tolerance": Specify convergence criteria. Run recycles until the distance between recycles is within the given tolerance value.
"stop_at_score": Compute models until pLDDT (single chain) or pTM-score (multimer) > threshold is reached. This speeds up prediction by running less models for easier queries.
"disable_cluster_profile": Experimental: For multimer models, disable cluster profiles.

Sets of parameters used for the massive sampling generation for H1140 with AFmassive:

Set 1 (Figure 2d):
- 3 neural network (NN) versions, 5 NN models per version
- 5 predictions per NN model, totaling 75 predictions
- no dropout
- templates used
- recycling: 20 steps and early stop tolerance set to 0.5

Set 2 (Figures 2a, 2b, 2e and Supplementary Figure 3):
- 3 NN versions, 5 NN models per version
- 67 predictions per NN model, totaling 1005 predictions
- no dropout
- templates used
- recycling: 20 steps and early stop tolerance set to 0.5

Set 3 (Figures 2b and 2f):
- 3 NN versions, 5 NN models per version
- 67 predictions per NN model, totaling 1005 predictions
- dropout activated (for Evoformer and structure module)
- no templates
- recycling: 20 steps and early stop tolerance set to 0.5

Set 4 (Supplementary Table 1):
- NN version v1, one NN model, 10 predictions
- dropout activated (for Evoformer and structure module)
- no templates
- recycling: 1000 steps and early stop tolerance set to 0.5

Set 5 (Figure 2c and Supplementary Table 1):
- NN version v1, one NN model, 11 predictions
- dropout activated (for Evoformer and structure module)
- templates not used
- recycling: 1000 steps and early stop tolerance set to 0.1

Two additional sets of parameters were used with ColabFold as a prediction engine:

Set 6 (Supplementary Figure 4a):
- 3 NN versions, 5 NN models per version
- 5 predictions per NN model, totaling 75 predictions
- no dropout
- templates not used
- recycling: 20 steps and early stop tolerance set to 0.5

Set 7 (Supplementary Figure 4b):
- 3 NN versions, 5 NN models per version
- 67 predictions per NN model, totaling 1005 predictions
- dropout activated
- templates not used
- recycling: 20 steps and early stop tolerance set to 0.5

Supplementary Figures and Tables


```
{
    "dropout_rate_msa_row_attention_with_pair_bias": 0.15,
    "dropout_rate_msa_column_attention": 0.0,
    "dropout_rate_msa_transition": 0.0,
    "dropout_rate_outer_product_mean": 0.0,
    "dropout_rate_triangle_attention_starting_node": 0.25,
    "dropout_rate_triangle_attention_ending_node": 0.25,
    "dropout_rate_triangle_multiplication_outgoing": 0.25,
    "dropout_rate_triangle_multiplication_incoming": 0.25,
    "dropout_rate_pair_transition": 0.0,
    "dropout_rate_structure_module": 0.1
}
```


Supplementary Figure 1: List of dropout rates of the Evoformer and of the structure module (last entry). A value between 0 and 1 is assigned to each dropout rate.

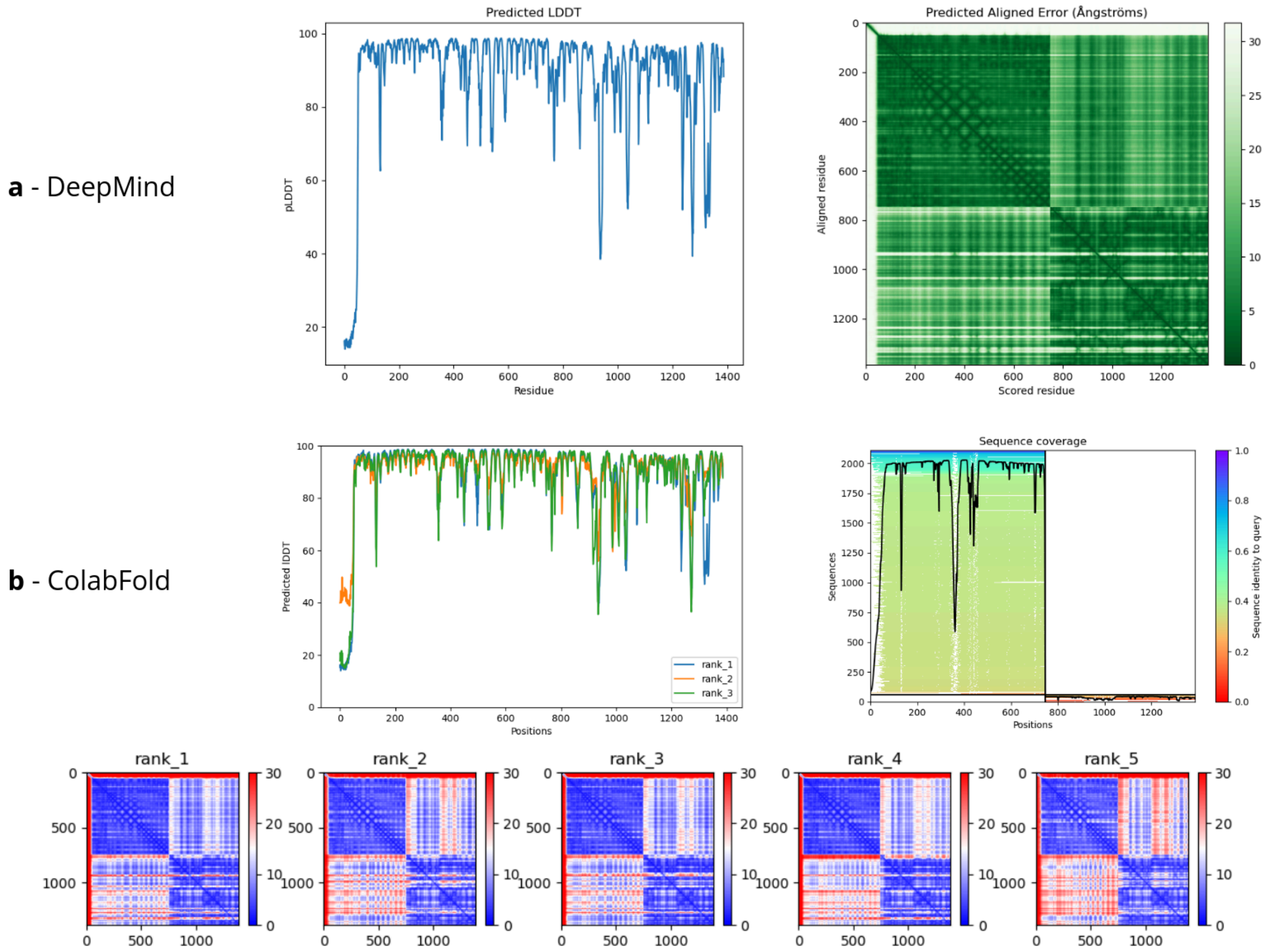

Supplementary Figure 2: pLDDT and Predicted Aligned Error plots. (**a**) DeepMind style (one of each per predicted structure) and (**b**) ColabFold style (multiple graphs in the same plot, left for pLDDT and bottom for Predicted Aligned Error); the ColabFold plots also show the sequence coverage (right-hand plot).

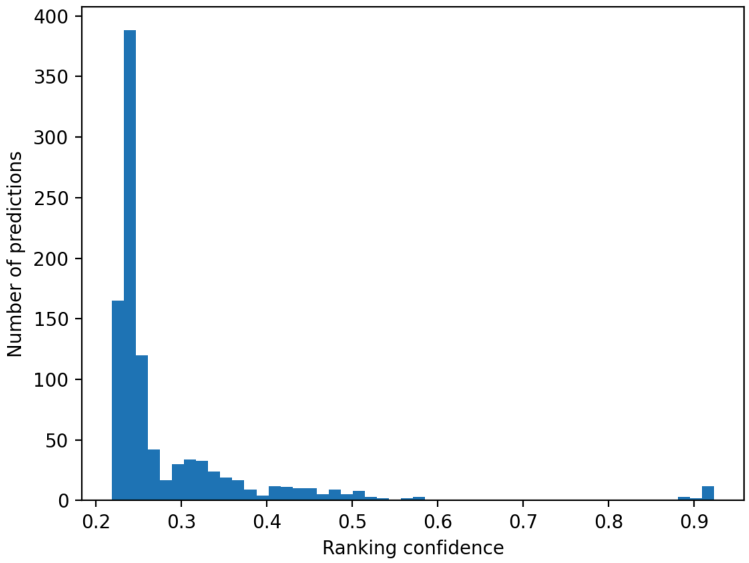

Supplementary Figure 3: Distribution of AlphaFold confidence scores for a prediction run of 1005 structures for CASP15 target H1140, running 67 predictions per NN model, with default parameters, *i.e.* without diversity parameters activated. AlphaFold confidence scores range from 0 to 1.

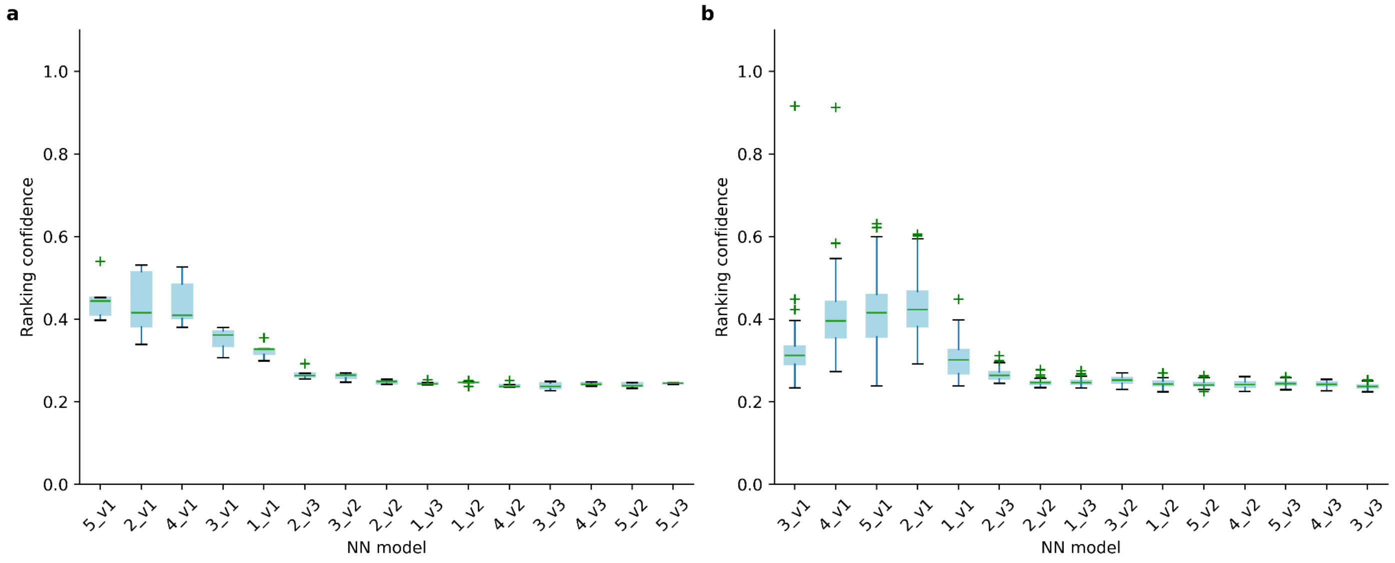

Supplementary Figure 4: Boxplots of the ranking confidence for each neural network model generated by MassiveFold using ColabFold for structure prediction for CASP15 target H1140. Each prediction run was parameterized without templates, 20 recycles and early stop tolerance set to 0.5 (**a**) computing 75 predictions without dropout activated, (**b**) computing 1005 predictions with dropout activated; each box in cyan extends from the first quartile to the third quartile, with a green line at the median, the whiskers reach out to the furthest data point within 1.5 times the interquartile range from the box and outliers (green crosses) lie beyond the whiskers.

Early stop tolerance	0.5	0.1
**Confidence Scores**	0.922	0.923
	0.920	0.923
	0.919	0.921
	0.917	0.921
	0.358	0.921
	0.245	0.921
	0.240	0.920
	0.234	0.920
	0.233	0.919
	0.189	0.918

Supplementary Table 1: Comparison of scores between 10 predictions for CASP15 target H1140. Each prediction run used the first neural network v1, dropout activated in the Evoformer and structure modules, without templates, with up to 1000 recycles, but a different early stop tolerance threshold (0.5 or 0.1).

Target	Top-1 DockQ Massive sampling	Top-1 DockQ AlphaFold3
**H1129**	**0.647**	0.059
**H1140**	0.819	**0.849**
**H1141**	**0.823**	0.030
**H1144**	**0.884**	0.317
**T1173**	**0.907**	0.043
**T1187**	**0.892**	0.102
**H1167**	0.064	**0.163**
**H1168**	0.549	**0.632**

Supplementary Table 2: Top-1 DockQ score among best 5 for 8 CASP15 targets. The targets were selected from [1] (values obtained from [2]) compared to the Top-1 DockQ score of the 5 AlphaFold3 predictions. In bold is highlighted the highest DockQ for each target; the two last rows are antibody-antigen targets.

References

1. Wallner, B. Improved multimer prediction using massive sampling with AlphaFold in CASP15. *Proteins* (2023) doi:10.1002/prot.26562.
2. Lensink, M. F. *et al.* Impact of AlphaFold on structure prediction of protein complexes: The CASP15-CAPRI experiment. *Proteins* **91**, 1658–1683 (2023).
